# A systematic review on the use of waste foundry sand as a partial replacement of common sand in mortars

**DOI:** 10.1007/s11356-026-38055-6

**Published:** 2026-07-18

**Authors:** Camila Rodrigues Moura, Gisleiva Cristina dos Santos Ferreira, Ivair Jose Sbroio, Daniel Augusto Oliveira Massolla

**Affiliations:** 1 Department of Architecture and Construction, School of Civil Engineering, Architecture and Urban Design, University of Campinas (UNICAMP), Campinas, Brazil; 2 Graduate Program in Technology, School of Technology, University of Campinas (UNICAMP), Limeira, Brazil

**Keywords:** Industrial waste, Mortar mixture, Mechanical properties, Chemical composition, Circular economy, Cementitious materials

## Abstract

The scarcity of natural resources worldwide has encouraged the reuse of industrial waste in cementitious materials. In this context, waste foundry sand (WFS) is a promising material for replacing common sand (CS) in non-structural mortars. This study reviews the literature on mortars containing WFS through a structured search and selection process. The review evaluates the mechanical properties of mortars considering WFS characteristics, mix proportions, specimen geometry, and workability conditions, while also discussing sustainability, circular economy benefits, and potential environmental risks. The reviewed literature revealed inconsistencies in the mechanical performance of mortars containing the same WFS replacement level. The reduction in compressive strength (CS) showed a low linear correlation with replacement level (*R*^2^ = 0.63). WFS replacement levels between 10 and 25% resulted in CS reductions ranging from approximately 2 to 26%, while higher replacement levels (30%, 50%, and 100%) led to reductions of up to 75%. Furthermore, many studies employed unusual mortar mix designs and failed to evaluate important properties for rendering mortars, such as modulus of elasticity and adhesion strength. Incomplete reporting of experimental parameters also hinders comparisons among studies and contributes to the inconsistencies observed in the literature. Although WFS pretreatment and calcination may enable higher replacement levels, further research is required to assess their compatibility with circular economy principles and their overall environmental benefits.

**Graphical Abstract:**

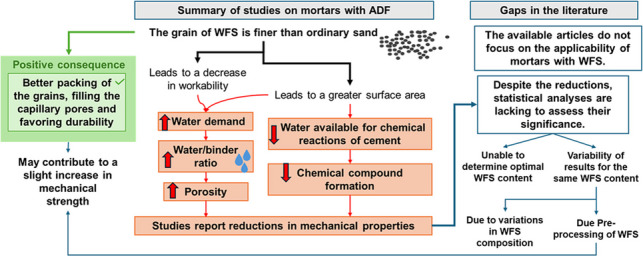

## Introduction

Currently, all productive sectors seek solutions to the environmental impacts resulting from their activities, which physically, chemically, or biologically transform the environment. According to John (2017), the extraction of non-renewable natural resources generates waste that exceeds 10 tons per person annually, causing negative impacts on the environment and society. Focusing on the construction sector, it is evident that this sector accounts for more than 50% of the planet's natural resource consumption, generating about 500 kg per person per year in waste.

In this context, there is a need to reduce the generation of waste and transform those that are compulsory into co-products. This is justified by the quality of most of the waste discarded in landfills, which has potential for reuse, as well as the fact that it increases the service life of landfills. Moreover, this new waste management format meets the concepts of the circular economy (Alves et al. 2014; Siddique et al. 2019) .

Waste foundry sand (WFS), which results from casting processes of metal parts, stands out among the most promising industrial solid waste to be directed as a construction material. This is justified by the physicochemical characteristics and the volume available, both in the national and international scenarios. There are several types of WFS, and the one from the “green sand” or “molding sand” is identified as the most appropriate due to its environmental classification (non-hazardous and non-inert—class II-A), according to NBR 10004 (ABNT 2024). In addition, there are documents, such as Chapter NR 538 and its guidance document issued by the Wisconsin Department of Natural Resources (2022) that encourage the beneficial use of industrial byproducts such as WFS. Thus, there is a growing interest from both academia and the productive sectors in the possibilities of using WFS, resulting in a significant increase in research on the topic (Na et al. 2015; Lee et al. 2016; Mobili et al. 2021; Silva et al. 2023; García et al. 2024).

In this context, it should be noted that mortar, which is in high demand worldwide, is an excellent material for meeting the requirements of the circular economy because it permits the use of large volumes of WFS to replace ordinary sand, especially in non-structural applications. However, existing review studies on the use of waste foundry sand (WFS) in cementitious materials have predominantly focused on the mechanical behavior of concrete (Mehta 2023, 2024; Sagar et al. 2025). Although a recent systematic review published in 2024 (García et al. 2024) included both mortars and concretes and demonstrated a linear correlation between WFS content and compressive strength, the analysis does not provide a detailed discussion of the strength reductions observed at each replacement level, nor of the factors responsible for the differences in mechanical behavior reported across studies.

These factors include specimen geometry, variations in mix composition, physicochemical properties of the material, and different curing conditions. In addition, the study does not provide specific recommendations for future research. Consequently, a gap remains in the literature regarding the relationship between these parameters and the variations in compressive strength observed at the same WFS replacement level.

Therefore, this study is aimed at conducting a systematic literature review to better organize and understand the properties and behavior of cement mortars containing WFS. The review seeks to identify the reductions in mechanical strength associated with different WFS replacement levels, as well as to discuss the main factors that hinder the definition of an optimal WFS content. Furthermore, based on the gaps identified in the literature, particularly regarding mechanical properties, this study proposes directions for future research. These contributions are expected to enable more consistent comparisons among future studies and support the advancement of WFS application in conventional cement mortars.

## Methodology and data of the systematic literature review

This section presents the methodology adopted for the systematic literature review on cement mortars containing waste foundry sand (WFS), enabling the achievement of the proposed objectives.

The methodology was conducted following the PRISMA (Preferred Reporting Items for Systematic Reviews and Meta-Analyses) guidelines. The bibliographic search was carried out using the Scopus and ScienceDirect databases, considering publications from 2010 to 2024. The search strategy employed the keywords “Mortar” and “Waste Foundry Sand”, combined using the Boolean operator AND.

The search resulted in the identification of 250 records. During the screening stage, 5 duplicate records were identified and removed, resulting in 245 unique studies for title and abstract screening. Subsequently, the studies were evaluated according to predefined inclusion and exclusion criteria.

The inclusion criteria were established to ensure the scientific consistency of the studies analyzed. Eligible studies included peer-reviewed journal articles, studies containing experimental data and/or systematic reviews, book chapters, and theses.

The exclusion criteria were applied to eliminate studies that did not fit the scope of this review. Therefore, studies containing the term “alkali-activated” in their titles or abstracts were excluded, as well as studies that did not provide relevant information for the objectives of this research.

After the title and abstract screening, 71 articles were selected for full-text assessment. At this stage, priority was given to studies investigating the mechanical properties of mortars produced with waste foundry sand. At the end of the eligibility process, 26 articles fully met the established criteria and were included in the review article.

**Fig. 1:**
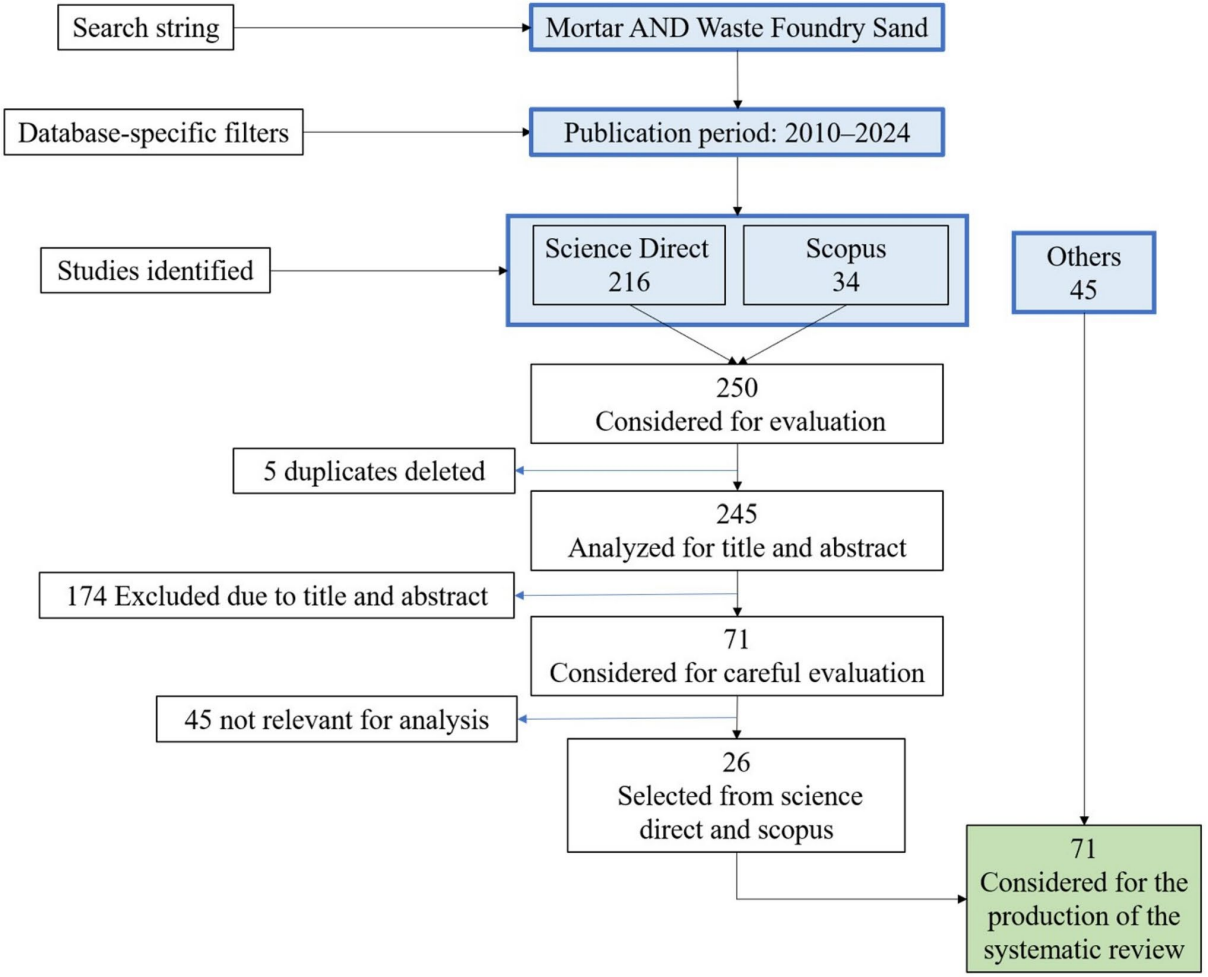
Representative flowchart of the steps carried out for the search and selection of publications relevant to the theme, according to systematic literature review (PRISMA method)

Additionally, documents from other sources, including technical standards, books, and studies identified through the reference lists of the selected articles, were also considered due to their relevance to the understanding of the topic. As a result, the final set of analyzed documents comprised 70 studies. The stages of identification, screening, eligibility, and inclusion are presented in Fig. 1.

**Fig. 2:**
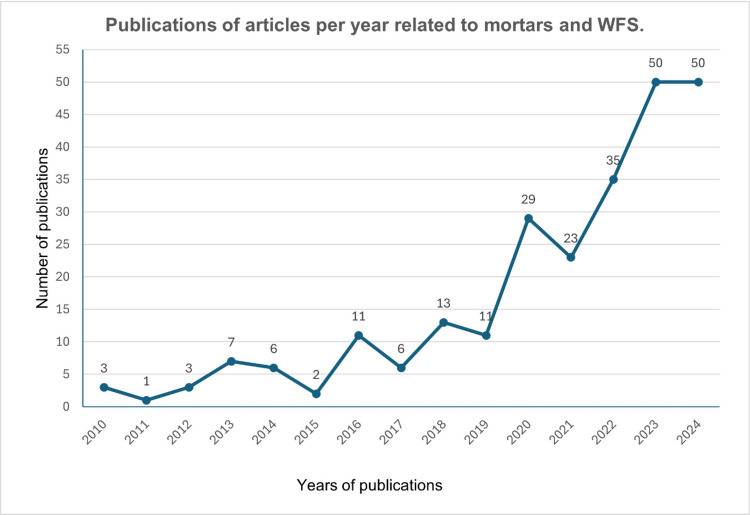
Total number of publications found per year in Science Direct and Scopus

The first analysis of the selected articles resulted in Fig. 2, which illustrates a significant increase in the number of publications on mortars containing WFS between 2010 and 2024, according to their annual distribution.

Although the number of publications related to mortars with WFS has increased, there are still few studies (26 publications) that focus on the mechanical properties of mortars with this waste product.

## State of the art

### Generation and types of WFS

The production of waste by the foundry sector depends on the production processes of the metal parts and the raw material. With this, there is the generation of various types of solid waste (exhaust and sweeping dust, WFS, and slags). When highlighting WFS, this material is responsible for the largest volume generated in the metal casting industry (85% of the sector's solid waste generation) (Carnin et al. 2022). The waste casting can be divided into two groups: molding sand (green or black sand), composed of 85–95% silica, 0–12% bentonite, and 2–10% coal powder, plus 2–5% water; core molding or chemically bonded sand (cold box and shell), composed of 93–99% silica and 1–3% organic resins (FIRST 2004). In the literature, some publications identify the molding sand with the following technical terms: waste foundry sand (WFS), foundry sand (FS), spent foundry sand (SFS), and green sand.

**Fig. 3:**
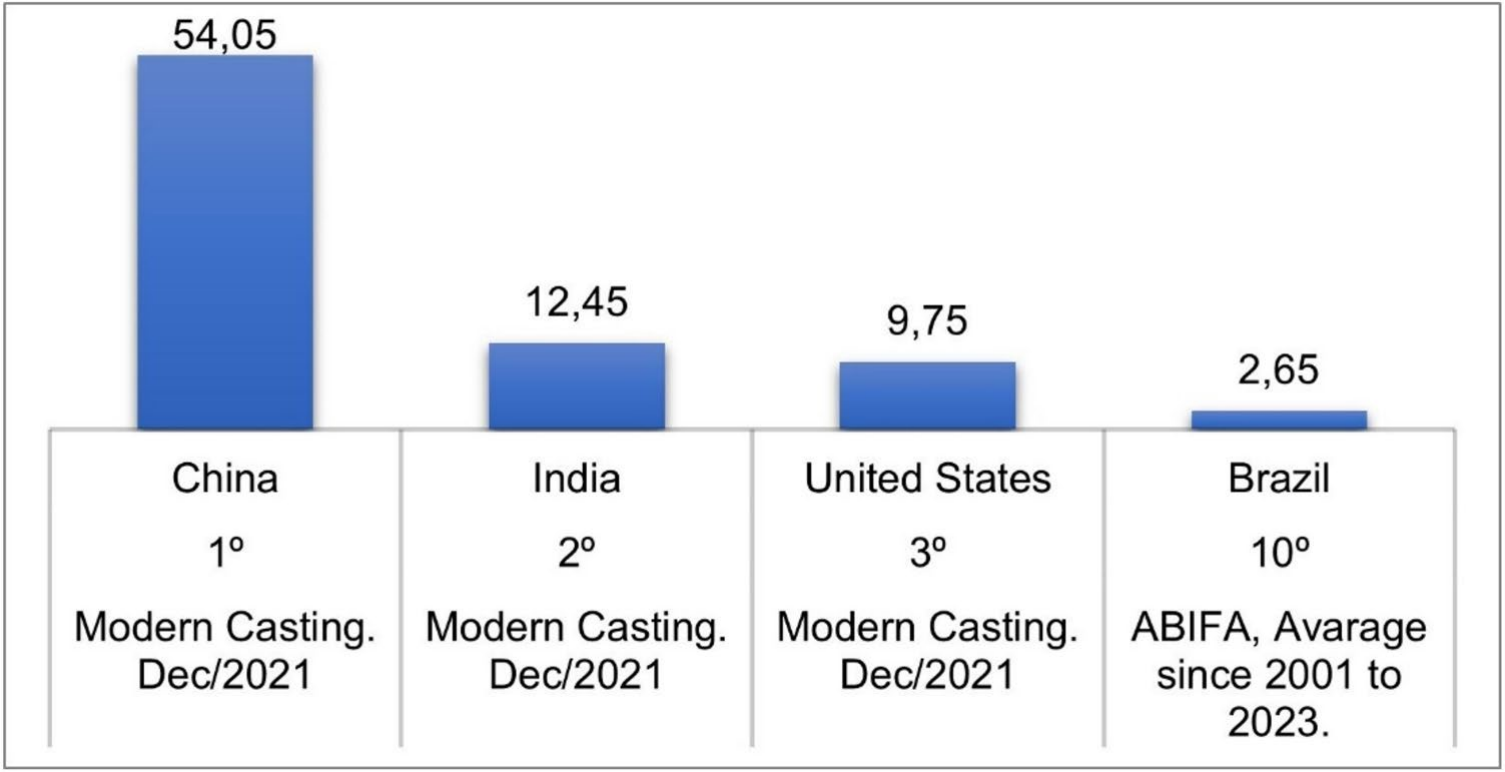
World production of castings

Considering that for each ton of steel production, 1 t of WFS is generated, it is estimated that only in Brazil, there is a generation of 3 million metric tons of WFS annually (ABIFA, 2023). Internationally, the three main countries generate approximately 70 million metric tons annually (54.05 million metric tons in China, 12.45 million metric tons in India, and 9.75 million metric tons in the USA), according to data released by Modern Casting (Modern Casting 2021), shown in Fig. 3.

WFS consists predominantly of silica and is used in metal casting processes until it becomes unsuitable for further application, due to physical and mechanical alterations in the grains that compromise casting quality, including abrasion and exposure to elevated metal temperatures (Siddique et al. 2019). When this occurs, there are additions of sand in the casting process, which results in the removal of part of the sand that has already gone through several casting cycles, which is identified as WFS (Carnin et al. 2022). It is worth mentioning that there are differences between WFS batches from different industries or even in the same one, depending on the stage of the process in which the disposal occurs (directly from the mold or by aspiration during the crushing of the molds) (Monosi et al. 2013).

### Characteristics of WFS

WFS stands out both for the volume generated and for the high potential for reuse in the civil construction area, due to physicochemical properties that are similar to those of common sand.

Therefore, WFS batches must be classified according to NBR 10004 (ABNT 2024), which makes it feasible to send them to controlled landfills or even a new use (Cioli et al. 2022). Thus, the chemical characterization of WFS batches is essential to enable the correct disposal or even use in other productive sectors. Table 1 was prepared based on the data presented in the studies selected for this literature review.

**Table 1:** Chemical composition of WFS samples, according to XRF analysis data obtained in the literature with a systematic literature review

Oxides	Authors								
	Filho et al. (2022)	Paul et al. (2021)	Sebki et al. (2019)	Vázquez-Rodriguez et al. (2018)	Çevik et al. (2017)	Navarro-Blasco et al. (2013)	Singh and Siddique (2012)	Guney et al. 2010)	Khanduri (2010)
SiO_2_	90.0	84	60.76	89.5	86.32	88.5	83.8	98	87.91
Al_2_O_3_	2.71	3.1	11.69	6.1	1.59	2.7 ± 0.3	0.81	0.8	4.70
Fe_2_O_3_	2.37	3.6	9.64	2.1	1.89	1.33 ± 0.02	5.39	0.25	0.94
Na_2_O	-	-	0.010	0.2	0.09	0.46 ± 0.06	0.87	0.04	0.19
CaO	0.23	0.3	6.33	0.9	0.17	0.16 ± 0.06	1.42	0.035	0.14
MgO	0.33	0.7	2.19	0.2	3.56	0.19 ± 0.04	0.86	0.023	0.30
K_2_O	0.45	0.4	1.28	0.1	0.31	0.77 ± 0.07	1.14	0.04	0.25
TiO_2_	-	0.1	-	-	-	0.61 ± 0.02	0.22	-	0.15
Cr_2_O_3_	1.25	4.6	-	-	-	0.045 ± 0.002	-	-	-
MnO	-	-	-	-	-	0.014 ± 0.007	0.05	0.01	0.02
SO_3_	0.18	-	1.85	-	-	-	0.21	0.01	0.09
NiO	-	0.3	-	-	-	-	-	-	-
As_2_o_3_	-	0.1	-	-	-	-	-	-	-
Zro_2_	-	0.1	-	-	-	-	-	-	-
P_2_Os	-	-	0.010	-	-	-	-	-	-
Mn_2_O_3_	-	-	-	-	-	-	-	0.01	0.02
Mn_3_O_4_	-	-	-	-	-	-	0.047	-	
SrO	-	-	-	-	-	-	-	-	0.03
Trace elements	-	-	-	-	-	-	-	0.836	
LOI*	1.93	1.6	-	-	-	-	-	4.3	5.15

*LOI: loss on ignition

The SiO_2_ content varies between 61 and 90% due to the origin of the raw material (quartz sand). Other oxides (Al_2_O_3_, Fe_2_O_3_, and MgO), on the other hand, come from the type of part and metal used in the molding process or are even necessary additions to the molding. This can be proven, for example, by the batch of WFS used by Vázquez-Rodriguez et al. (2018), which came from the manufacture of aluminum parts and presented 6% Al_2_O_3_. Although Sebki et al. (2019) did not report the type of molding that gave rise to the batch they studied, they also used a batch of WFS with a high content of aluminum oxides (11.69%).

Furthermore, according to what is presented in the literature (Khanduri 2010; Guney et al. 2010; Singh and Siddique 2012; Sebki et al. 2019; Paul et al. 2021; Filho et al. 2022; Massolla et al. 2024), both the real specific mass and the unit mass of WFS have lower values compared to common sand. In the case of unit mass, this behavior can be justified due to the volume of voids contained between the grains. Regarding the specific mass, this occurs due to the chemical compounds, such as those that are added in the “green sand” molding process (coal powder and bentonite) (Candian Filho et al. 2022). It should be noted that these variations can be beneficial depending on the type of application that each batch of WFS can be used.

When highlighting the characteristics of WFS batches, related to granulometry, there are significant differences that are justified by the functionality and performance for each type of part and metal alloy used during the molding process. Deng and Tikalsky (2008) report that the molding of aluminum parts occurs with finer sands (better finish). Coarser sands, on the other hand, are ideal for the steel foundry process, as they facilitate the release of gases. Fiore and Zanetti (2007), when studying the main types of WFS, classified them into three categories (≤ 0.1 mm, 0.01 to 0.6 mm, and > 0.6 mm). In this sense, Navarro-Blasco et al. (2013) analyzed the WFS granulometry for mortar production, in which 60% and 30% of the grains were around 0.25 and 0.5 mm, respectively (Feijoo et al. 2021); when studying another batch of WFS, also for mortar production, the report states that 85% of the material was comprised between 0.15 and 0.6 mm.

**Fig. 4:**
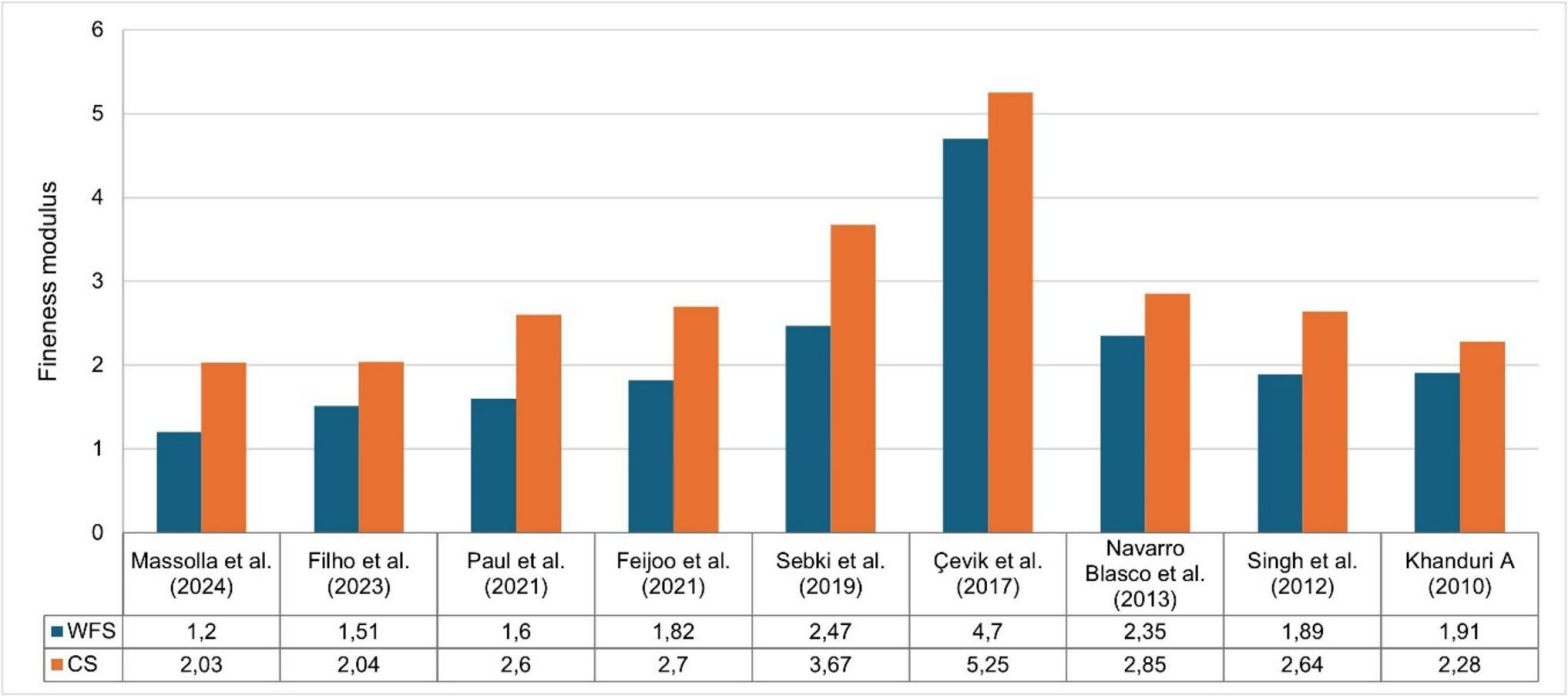
Fineness modulus of waste foundry sand (WFS) and common sand (CS)

Therefore, to analyze these grain size variations, it is interesting to identify the fineness modulus (FM) and compare it to those obtained for common sand. To assist, Fig. 4 was elaborated with such information, based on the data available in the literature consulted.

The fineness modulus values of the WFS batches ranged between 1.5 and 2.0. When compared to the values of ordinary sand (2.0 to 5.3), one can conclude that the differences are significant, which should be considered in the case of directing WFS to applications in other productive sectors.

In this context, the numerous possibilities of using WFS in civil construction stand out, from the granulometric stabilization of soils for geotechnical works (dams and landfills) and paving (structural layers and asphalt mixtures) to cement materials (concretes, mortars, and prefabricated).

In addition, it is understood that the activities of the foundry sector are responsible for direct environmental impacts (consumption of natural resources and generation of waste), as well as indirect, such as possible contamination (air, soil, and water) and reduction of the service life of landfills (Tittarelli 2018; Bochare et al. 2024). Therefore, the use of WFS in civil construction is very promising, both in the scope of supply and demand, as well as regarding physical and chemical aspects (Navarro-Blasco et al. 2013; Çevik et al. 2017; Srivastava and Sing 2020; Singh et al. 2021; Sithole et al. 2022).

### Applications of WFS in cement materials

There are several studies on the use of WFS in civil construction that focus on paving and geotechnical works (asphalt mixtures, soil improvement for base and sub-base) (Dyer et al. 2021; Magalhães et al. 2023; Gambalonga et al. 2023; Machado et al. 2023; Godoi et al. 2023; Alo and Oyedepo 2024; Aguiar et al. 2024). On the other hand, there are also studies on the use of WFS as a fine aggregate in cement materials (concretes, concrete artifacts, and mortars) (Srivastava and Sing 2020; Singh and Dhiman 2021; Paiva et al. 2021; Paul et al. 2022).

According to the literature, the use of WFS in cement materials occurs mainly in partial or total replacement of common sand in concrete (Salim et al. 2017; Sravani et al. 2018; Sangavi and Angu Senthil 2019; Singh and Dhiman 2021; Ramkumar et al. 2022; García Del Angel et al. 2023). Some authors claim that the alkalinity of the cement matrix can immobilize contaminants, for example, heavy metals present in some types of waste (Ahmad et al. 2022; Chifflard et al. 2024). This would be an advantage regarding the destination of WFS in concrete and other cement materials, as it reduces the risks of contamination with leachates from these materials.

Concerning the use of WFS as an aggregate in concrete artifacts, the greatest technological control during production stands out, which can result in construction elements with greater strength than conventional materials. This contributes to reducing the environmental impacts of civil construction (Rajemahadik et al. 2014; Mehta et al. 2018; Nyembwe et al. 2018; Sebki et al. 2019; Sabour et al. 2022; García Del Angel et al. 2023).

However, as already described, variations in the chemical composition of WFS batches can influence the hydration of Portland cement, positively or negatively. Monosi et al. (2013) conducted a study with cement pastes produced with water from the washing of two types of WFS. Water with a higher content of alkaline ions resulted in a paste with a higher Ca(OH)2 content in the first hours of hydration (2 to 5 h). Therefore, they concluded that the addition of WFS can cause an accelerating effect on cement hydration if its soluble alkali ion content is particularly high.

Sangavi and Angu Senthil (2019) conducted a systematic literature review on the use of WFS in concretes. From the analysis of the results found in the literature, these authors concluded that the production of concrete with substitution contents between 20 and 40% did not present significant changes in mechanical properties.

Siddique and Singh (2011) studied contents from 5 to 15% of WFS replacing common sand in concrete, and they found a reduction in workability. On the other hand, the authors claim that water absorption decreases with increasing WFS content (from 5 to 15%), which was justified due to the reduction of the void index and/or porosity compared to the standard trace, considering the effect of grain packing, obtained with the granulometric composition resulting from the mixture of common sand + WFS.

In highlighting the economic aspects, Gambalonga et al. (2023) used as an example, a foundry industry responsible for the generation of 4800 t/year of WFS. The authors identified the costs involved with logistics of destination of this waste (US$2.93/metric ton) and discard in industrial landfills (US$25.43/metric ton), which add up to US$136,128 t/year. Moreover, Siddique et al. (2019) reported that the annual destination of about 9 million metric tons of WFS to landfills in 2000 resulted in costs from US$135 million to US$675 million in the USA. To detail this cost, one can cite the data by Machado et al. (2023), who established a scenario of a foundry company that generates 400 t/month of WFS, which resulted in expenses of US$122,000/year (discard in landfill), in addition to US$14,000/year (transport logistics and intermediate storage). Therefore, enabling the use of WFS in civil construction can bring positive impacts on all aspects of sustainable production, based on circular economy concepts.

Nevertheless, when looking for data on the use of WFS in mortars, few studies are available in the literature compared to those on the use in concretes. Thus, we identified gaps in the knowledge about mortars with WFS, which directed the focus of this systematic review. In the following sections, the sustainability aspects of mortars produced with WFS and environmental impacts associated with WFS leachate, as well as the influence of curing time on their mechanical properties, will be discussed. Subsequently, a compilation of the main compressive strength (CS) and flexural strength (FS) results reported in the literature will be presented. Based on this analysis, the factors that may influence the mechanical performance of these mortars will be discussed, including fresh-state properties, specimen geometry and dimensions, the FM of the WFS, and the different curing methods employed.

### Sustainability

Regarding the environmental advantages of the use of WFS in cement materials, some authors concluded that the use of WFS as a raw material in civil construction allows a reduced exploitation of natural resources and, consequently, a reduction of greenhouse gas emissions (Ahmad et al. 2022; García Del Angel et al. 2023; Chifflard et al. 2024).

However, to have great advances in the use of WFS, it is of paramount importance that initiatives are taken together with environmental agencies, since individuality and lack of technical information are the main obstacles to the evolution of the use of this waste (Paiva et al. 2021). In this sense, Brazil opened paths for the employment of WFS in civil construction with the guidelines and instruments of the National Solid Waste Policy, Law no. 12,305/2010 (PNRS), which initially encourages the reuse and recycling of waste, putting the disposal in landfills as a last option.

In addition to the incentive of PNRS, the demands of society concerning the consumption of products and services with lower environmental impacts cause economic sectors to incorporate circular economy concepts in their activities; that is, production with less generation and waste disposal. Civil construction has also directed the management of its activities in this direction, including in its production, more sustainable technologies and products (Singh et al. 2021; Carnin and Moterani 2024).

In the same direction, the foundry sector of metal parts seeks a more sustainable destination for its waste, such as WFS. In addition to environmental impacts, there are those related to the cost of operation and destination (economic impacts). Thus, the union of interests of these two sectors has resulted in several studies and even in practical applications, with the use of WFS as raw material (Çevik et al. 2017; Singh et al. 2021; Feijoo et al. 2021; Sithole et al. 2022).

The analyzed studies demonstrate that the partial replacement of natural sand with waste foundry sand (WFS) can achieve replacement rates ranging from 20 to 50%, depending on the application and the required mechanical performance. In particular, Sagar et al. (2025) reported that concrete containing 30% WFS as a replacement for natural sand achieved a compressive strength of 37.11 MPa, representing an increase of approximately 34% compared to the reference concrete. Similar findings were reported by Mehta et al. (2018), who identified optimal replacement ranges capable of maintaining or improving the mechanical properties of concrete.

From a circular economy perspective, these replacement levels represent a potential diversion of waste from landfills equivalent to 20–50% of the fine aggregate mass used in the mixtures, consequently reducing the demand for natural resources. Considering that natural sand is one of the main constituents of concrete, its partial replacement with WFS directly contributes to the conservation of non-renewable mineral resources and to the reduction of impacts associated with the extraction, processing, and transportation of natural aggregates.

Furthermore, the reviewed studies highlight that the reuse of WFS reduces the need for landfill disposal and decreases the demand for natural sand, thereby contributing to natural resource conservation and supporting circular economy strategies (Siddique and Kadri 2011; Cioli et al. 2022; Machado et al. 2023; Aguiar et al. 2024). From a theoretical life cycle perspective, the partial replacement of virgin raw materials with industrial waste also has the potential to reduce the environmental impacts associated with the extraction and transportation of natural aggregates (Ahmad et al. 2022; Machado et al. 2023).

Although the analyzed articles do not provide Life Cycle Assessment (LCA) results or detailed environmental inventories that would allow a precise quantification of carbon footprint reductions, the findings indicate a significant contribution to material circularity and to the mitigation of environmental impacts related to industrial waste management and the exploitation of natural resources.

Table 2 presents the quantitative indicators extracted from the reviewed studies on the incorporation of WFS into cementitious materials. The table consolidates the range of natural sand replacement by WFS reported in the analyzed studies (20–50%), the corresponding potential for landfill waste diversion, and the specific mechanical performance reported by S (Sagar et al. 2025) for the mixture containing 30% WFS (37.11 MPa; approximately 34% higher than the reference concrete). These indicators are presented as measures of circularity and resource conservation rather than as Life Cycle Assessment (LCA) results or direct carbon footprint reduction metrics.

**Table 2:** Quantitative and environmental indicators associated with the use of WFS in mortars and cementitious materials

Authors	Evaluated material	WFS content	Environmental quantitative indicator	Main environmental benefit
Siddique e Singh (2011)	Concrete containing WFS	10–30%	Up to 30% reduction in natural sand consumption	Conservation of mineral resources
Singh e Siddique (2012)	Concrete containing WFS	10–30%	Up to 30% potential diversion of industrial waste	Reduction of landfill disposal
Feijoo et al. (2021)	Mortars containing WFS	10–40%	Up to 40% replacement of natural aggregate	Conservation of natural resources
García Del Angel et al. (2023)	Mortars containing recycled foundry sand	10–50%	Up to 50% replacement of natural sand	Material circularity
Guney et al. (2010)	High strength concrete	10–50%	Up to 50% reuse of WFS	Reduction of waste sent to landfill
Monosi et al. (2013)	Mortars and concretes	10–30%	Reuse of industrial by-product	Reduced demand for virgin raw materials
Sebki et al. (2019)	Self-compacting cementitious mortars	10–30%	Simultaneous use as aggregate and cementitious material	Increased circularity
Sabour et al. (2022)	Geopolymer-based mortars	10–40%	Positive environmental assessment compared to conventional mixtures	Reduction of global environmental impacts
Cioli et al. (2022)	Scenarios for reuse and disposal	Not applicable	Environmentally superior reuse compared to landfill disposal	Reduction of the environmental burden of disposal
Machado et al. (2023)	Brazilian waste foundry sand	Not applicable	Analysis from a circular economy perspective	Valorization of industrial waste
Chifflard et al. (2024)	Construction materials	Not applicable	Feasibility of sustainable recycling of WFS	Sustainable resource management

The analysis of the reviewed studies demonstrates that the incorporation of waste foundry sand (WFS) into mortars and cement-based materials typically occurs at replacement levels ranging from 10 to 50% of natural sand. From an environmental perspective, these percentages represent an equivalent reduction in the demand for natural aggregates and the diversion of industrial waste from landfills. Furthermore, recent studies on environmental assessment and the circular economy indicate that the reuse of WFS exhibits superior environmental performance compared to conventional disposal practices, reinforcing its potential as a sustainable strategy for the construction sector (Cioli et al. 2022; Sabour et al. 2022; Machado et al. 2023).

### Environmental impacts associated with WFS leachate

Environmental impact assessment is a fundamental aspect for enabling the use of waste foundry sand (WFS) in mortars. In this context, the evaluation of leaching potential plays an important role, particularly in exterior rendering mortars, since contaminants eventually released from the material may migrate through the soil and reach groundwater resources. In a review focused on the environmental aspects of WFS reuse, Cioli et al. (2022) highlighted that the chemical composition of this residue varies significantly according to the cast metal, the binders employed, and the reuse history of the sand. The review identified chromium, nickel, and zinc as the most frequently detected metals, in addition to organic contaminants such as hydrocarbons, phenols, and naphthalene-derived compounds. Although several studies reported low mobility of these contaminants in leaching tests, the literature also documented cases in which nickel, chromium, zinc, fluorides, and dissolved organic carbon exceeded applicable environmental limits.

Overall, the available literature indicates that the contamination potential associated with WFS cannot be generalized, as its chemical and environmental characteristics depend on several factors related to the foundry process. Among the most relevant factors are the type of cast metal, the molding and core-making systems employed, the binders used, the constituents of the sand matrix, and the number of reuse cycles. Consequently, different WFS sources may exhibit distinct chemical compositions, influencing the occurrence, mobility, and potential ecotoxicological effects of inorganic and organic contaminants. In this regard, the literature emphasizes the need for waste-specific environmental characterization, avoiding generalized assumptions regarding environmental safety and supporting more reliable assessments for applications in cementitious materials (Alves et al. 2014; Cioli et al. 2022; Chifflard et al. 2024; Bochare et al. 2024; Alias et al. 2024).

Chifflard et al. (2024) investigated the chemical properties and reuse potential of waste foundry sands obtained from three German foundries. The authors reported that WFS may accumulate heavy metals, organic compounds, and other contaminants throughout successive reuse cycles during the foundry process, representing a potential source of soil and groundwater contamination. To assess this risk, extraction tests were performed according to the LAGA M20 guidelines, a German regulatory framework used to evaluate contaminant release from mineral wastes. The results showed that samples collected from more advanced stages of the reuse cycle exhibited elevated concentrations of chromium, copper, and nickel, exceeding the regulatory limits established for the raw material. However, after stabilization with 3% and 5% Portland cement (by mass), contaminant mobility was reduced, and the concentrations released during testing remained below the applicable environmental limits. Therefore, cement incorporation may act as a chemical stabilization mechanism, reducing the leaching potential of contaminants.

Bochare et al. (2024) evaluated the use of a foundry sand classified by the authors as Toxic Foundry Sand (TFS), obtained from an industrial cluster comprising approximately 50 foundries, as a partial replacement for natural sand in concrete. According to the authors, the material became “toxic” after successive reuse cycles in foundry operations, accumulating metallic residues and other contaminants. For the environmental assessment, a mixture containing 30% TFS and 70% natural fine aggregate was adopted. Unlike conventional leaching tests, the authors employed a procedure referred to as Toxic Foundry Sand Contact Water (TFSCW), obtained by immersing concrete specimens containing TFS in water for extended periods, simulating exposure to rainwater, curing water, or storage conditions. The analyses revealed significant changes in water quality, with pH, turbidity, chloride concentration, and alkalinity values exceeding the drinking water limits recommended by the World Health Organization (WHO). Although the incorporation of WFS into cementitious matrices represents a promising alternative for the reuse of industrial residues, prolonged contact with water may promote contaminant transfer or substantially alter its physicochemical characteristics, reinforcing the need for environmental monitoring and further assessment of the risks associated with the large-scale application of these materials.

Alias et al. (2024) performed an ecotoxicological assessment of 25 WFS samples collected from Italian foundries employing different molding systems, including green sand, phenolic resins, and other binder systems. The samples were subjected to leaching tests according to EN 12457-2. The authors also investigated the effects of the leachates on organisms representing different aquatic trophic levels, including the bioluminescent bacterium *Aliivibrio fischeri*, the microcrustacean *Daphnia magna*, and the microalga *Pseudokirchneriella subcapitata*. The results revealed substantial variability among the evaluated samples, with significant toxic effects observed for all tested organisms. The samples exhibiting the highest ecotoxicity were associated with steel foundries and showed elevated concentrations of copper, nickel, fluorides, and chemical oxygen demand (COD). The findings indicated that factors such as the cast metal, aggregate type (silica, chromite, or zircon), binder system, and sand reuse history significantly influence the chemical composition of the leachates and their biological effects.

Similarly, Alias et al. (2024) evaluated the metal leaching potential of 16 WFS samples obtained from 10 ferrous foundries located in Santa Catarina State, Brazil. The samples consisted predominantly of green sands used in molding operations, associated with core-making systems based on phenolic and polyurethane resins. The authors determined total metal concentrations and conducted leaching tests using the Toxicity Characteristic Leaching Procedure (TCLP), a widely adopted method for hazardous waste classification. The results indicated that none of the samples could be classified as hazardous waste, since contaminant concentrations in the leachates remained below the limits established by Brazilian regulations. However, when compared with groundwater quality standards, concentrations of aluminum, boron, barium, chromium, iron, mercury, manganese, nickel, lead, and zinc exceeded the respective guideline values in at least one sample. Based on these findings, the authors applied the U.S. EPA Industrial Waste Management Evaluation Model (IWEM) to simulate the transport of contaminants to groundwater under different climatic scenarios. The simulations indicated that the environmental impact potential is strongly dependent on local conditions, with the highest risks observed under scenarios characterized by high precipitation and low evaporation rates. The authors further noted that TCLP employs an acidic extraction solution designed to represent specific waste disposal conditions, whereas the IWEM scenarios incorporate additional environmental factors capable of influencing contaminant mobility. Therefore, it was concluded that the unconfined reuse of WFS may represent an environmentally viable alternative under a wide range of conditions, although materials with higher leaching potential and environmentally sensitive scenarios should be evaluated on a case-by-case basis before large-scale implementation.

### Curing time

With respect to the curing time after casting, a reduction may occur with an increase in the WFS content, according to the results of some authors (Monosi et al. 2013; Navarro-Blasco et al. 2013; Matos et al. 2019). Monosi et al. (2013), who produced mortars with washed WFS, reported that this procedure reduced the presence of alkaline ions, with no significant changes in curing time when compared to the reference mortar. Higher alkalinity is due to the presence of bentonite in some types of WFS, which assumes different functions during cement hydration processes. Bentonite absorbs water quickly, releasing a lot of heat and resulting in the acceleration of chemical reactions in the early stages. However, during hydration, there is an inversion in this behavior, as the bentonite clay minerals start a slow process of releasing the water absorbed initially. This can cause an excess of water in the paste, which slows down the reactions still in progress, in addition to damaging the aggregate/paste interface and increasing the porosity after drying and hardening of the mortar (Carasek 2017). It should be noted that, depending on the functionality of the mortar, the reduction in curing time may be favorable (Navarro-Blasco et al. 2013).

**Fig. 5:**
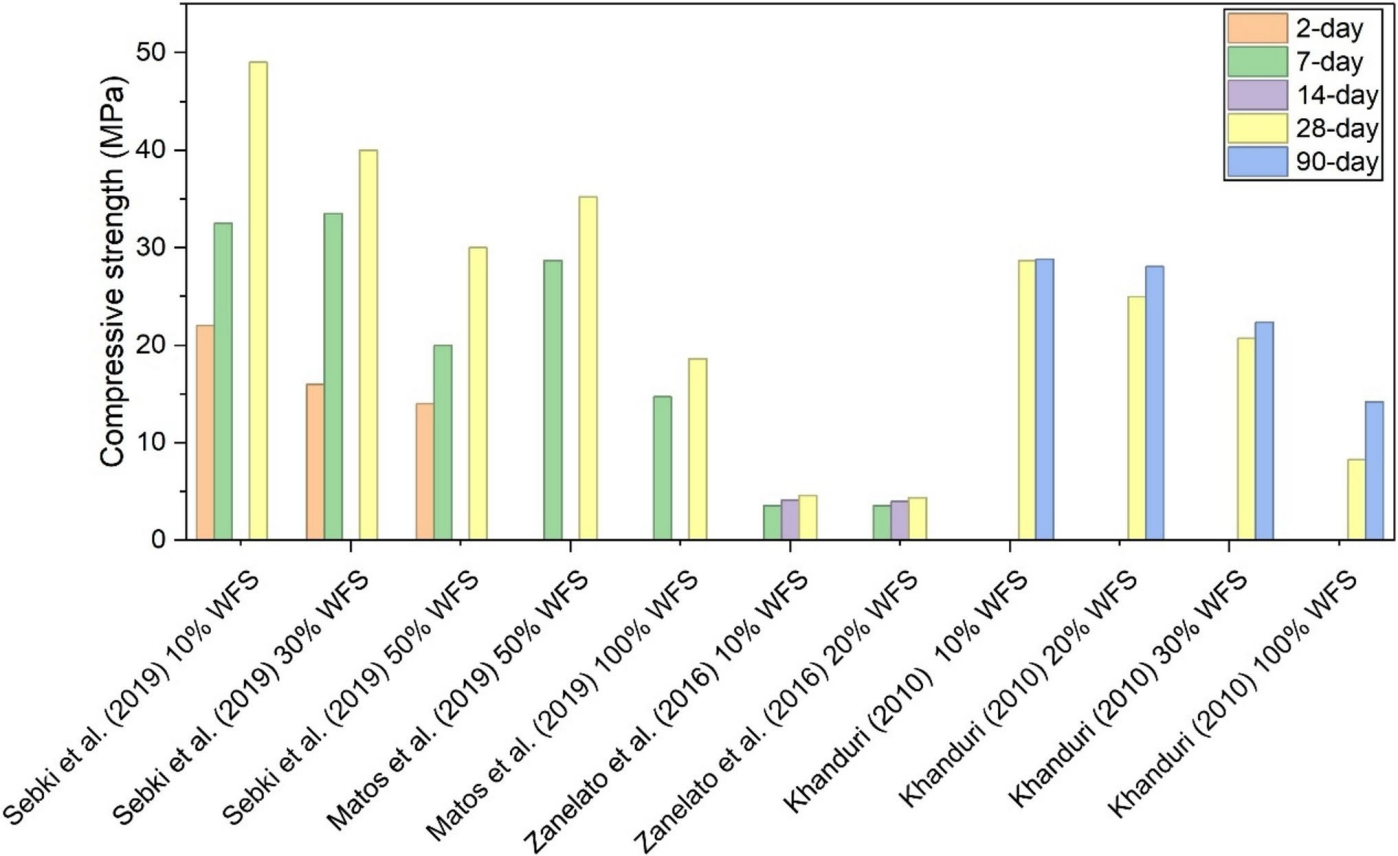
Behavior of cement mortars after 28 days of curing at different WFS levels: (**a**) compressive strength (CS); (**b**) flexural strength (FS)

Authors also reported that curing age (3, 7, 28, and 90 days) influences the mechanical properties of mortars containing WFS (Khanduri 2010; Zanelato et al. 2016; Sebki et al. 2019; Matos et al. 2019). Based on the studies identified in the literature, a subset of authors who used waste foundry sand (WFS) from molding processes (green sand) was selected. Using these data, the graph presented in Fig. 5 was developed, showing the compressive strength (CS) values as a function of curing time for different authors and various levels of natural sand replacement by WFS.

Despite the variations in compressive strength values reported by different authors and for different WFS replacement levels, the results presented in Fig. 5 show a consistent increase in compressive strength with curing time. This trend was observed in all studies analyzed and reflects the continued hydration of cementitious compounds over time. In this sense, Khanduri (2010) also reports that a mortar with WFS has lower reductions in mechanical properties, after 90 days of curing, compared to a mortar without WFS, because at long stages, almost all chemical compounds responsible for mechanical resistance have been formed, maintaining stability of these resistances. Therefore, although the initial compressive strength suffers a drop, this reduction becomes less significant in advanced stages of curing, indicating an improvement in mechanical performance over time.

Regarding curing time, it is noteworthy that only a limited number of studies have evaluated mortars containing WFS at advanced ages, particularly at 90 days. This limitation, combined with the small number of authors employing conventional or comparable mix proportions, makes it difficult to perform comprehensive assessments of the long-term performance and durability of these mortars. Nevertheless, some researchers have conducted 90-day evaluations and investigated durability-related properties (Khanduri 2010; Sabour et al. 2022). In this context, a recent systematic review on the subject compiled and analyzed the main studies available in the literature, including several authors also discussed in the present review, highlighting results related to effective porosity, abrasion resistance, and resistance to sulphate attack (García et al. 2024).

## Summary of mix designs and properties of mortars containing WFS

By specifying the type of mortar for the application, one has coating mortars. The most important mechanical properties of this type of mortar are flexural strength (FS), adhesion strength, and static modulus of elasticity (E). This is justified due to the movements related to temperature and humidity variations, which occur differently for the substrate and mortar, because of the differences between the thermal properties of each material (Gaibor et al. 2023). Adhesion strength, obtained from the test to determine the adhesion potential of the mortars to the substrate, provides essential information to verify the performance of coating mortars. However, there is little data available in the literature on the subject, because most studies investigate the behavior of the waste and not the functionality of mortars with waste.

To systematically organize the mechanical property data reported in the literature, Table 3 was compiled, including information on WFS replacement levels, mix proportions, compressive strength (CS), and flexural strength (FS). Only results obtained after 28 days of curing were considered, as curing age significantly influences the mechanical performance of mortars, as discussed in the previous section. The 28-day curing period was selected because it is the most commonly adopted age in the literature and corresponds to the standard curing period specified by most codes and standards for the evaluation of cementitious materials. Furthermore, only cement-based mortars were included in the analysis. The focus on compressive and flexural strength was motivated by the fact that these are the mechanical properties most frequently reported in studies investigating the incorporation of WFS into mortar mixtures.

**Table 3:** Summary of studies on mortars containing waste foundry sand (WFS), including WFS type, mortar formulation (mf), specimen geometry, WFS content, water-to-cement ratio (W/C ratio), consistency (Cons.), compressive strength (CS), and flexural strength (FS), all measured after 28 days of curing

Authors	mf (mass)	WFS type	Specimen geometry	WFS content (%)	CS (MPa)	FS (MPa)	W/C ratio	Cons. (mm)
Massolla et al. (2024)	1:6	Green untreated	Prismatic 40 × 40 × 160 mm^3^	0	18.85	4.47	0.68	261
				30	17.71	3.92	0.71	261
				50	13.78	3.21	0.75	262
				100	10.18	3.21	0.81	261
García Del Angel et al. (2023)	-	Green untreated	Prismatic 40 × 40 × 160 mm^3^	0	~ 59	~ 6.4	0.5	~ 15.5
				25	~ 56	~ 6.5	0.51	
				50	~ 46	~ 5.6	0.57	
				75	~ 43	~ 5.8	0.62	
				100	~ 37	~ 4.9	0.70	
Paul et al. (2022)	1:3	Chemical treated (method not specified)	Prismatic 40 × 40 × 160 mm^3^	0	58.7 ± 1.4	7.0 ± 0.4	0.50	35 ± 1
				10	54.5 ± 1.0	6.4 ± 0.6	0.45	33 ± 1
				30	49.3 ± 1.0	5.5 ± 0.5	0.48	38 ± 1
				50	42.6 ± 1.3	5.4 ± 0.1	0.50	38 ± 1
				80	31.3 ± 1.9	4.8 ± 0.2	0.56	34 ± 0
				100	25.1 ± 1.6	4.0 ± 0.9	0.62	34 ± 0
Sabour et al. (2022)	1:2.75	Chemical untreated	Cubic 50 mm^3^	100	10	-	0.49	-
Feijoo et al. (2021)	1:2.75	Green untreated	50 × 50 × 50 mm^3^	0	43.73	-	0.5	-
				15	42.88	-		
				20	41.95	-		
				25	39.11	-		
Thamaraiselvi et al. (2020)	1:4.53	Not specified	-	0	~ 34^*^	~ 4.64^*^	-	~ 160
				10	~ 41^*^	~ 4.68^*^		~ 180
				20	~ 39^*^	~ 4.72^*^		~ 190
				30	~ 36.5^*^	~ 4.74^*^		~ 185
Sebki et al. (2019)	1:2	Green untreated	Cubic 50 mm^3^ to Rc Prismatic 40 × 40 × 160 mm^3^ to FS	0	~ 50.5	~ 9.8	0.351	~ 205
				10	~ 49	~ 9.6		~ 200
				30	~ 40	~ 8.4		~ 190
				50	~ 30	~ 6.3		~ 180
Matos et al. (2019)	1:3	Green untreated	Cylindrical 50 × 100 mm	0	41.8	-	0.55	255
				50	35.2	-		200
				100	18.6	-		155
Vázquez-Rodriguez et al. (2018)	1:2.75	Chemical untreated	Cubic 50 mm^3^	0	25	-	0.485	110 ± 5
				100	6	-		-
Çevik et al. (2017)	1:3	Chemical untreated	Cubic 40 mm^3^	0	35.04	-	0.5	-
				15	33.13	-		
				30	~ 31.5	-		
				45	~ 26	-		
				60	~ 24.9	-		
Zanelato et al. (2016)	1:3	Green untreated	Prismatic 40 × 40 × 160 mm3	0	4.30	1.30	0.69	263
				10	4.59	1.51	0.69	256
				20	4.32	1.29	0.78	256
Navarro-Blasco et al. (2013)	1:1.56	Not specified	Cylindrical 40 mm height 37 mm diameter	50	~ 14	-	0.37	144
Monosi et al. (2013)	1:3	Green—mould disposal (untreated)	Prismatic 40 × 40 × 160 mm	0	48	-	0.5	145
				20	39	-		115
				30	36	-		110
Khanduri (2010)	1:3	Green untreated	Cubic 70.9 mm	0	33.58	-	0.46^a^ or 0.54^b^	-
				10	28.67	-		
				20	24.97	-		
				30	20.71	-		
				40	21.13	-		
				60	17.07	-		
				100	8.23	-		

*Value of mortar with WFS that used additions in cement

^a^Value obtained from the author’s table

^b^Value reported in the author’s text

It is important to note that some authors do not provide exact numerical values for compressive strength (CS) and flexural strength (FS), as the results are reported only in graphical form. In such cases, the values were estimated through visual interpretation of the published figures. Due to the inherent limitations of this procedure, some results are indicated with the approximation symbol (~) to reflect the uncertainty associated with their determination.

**Fig. 6:**
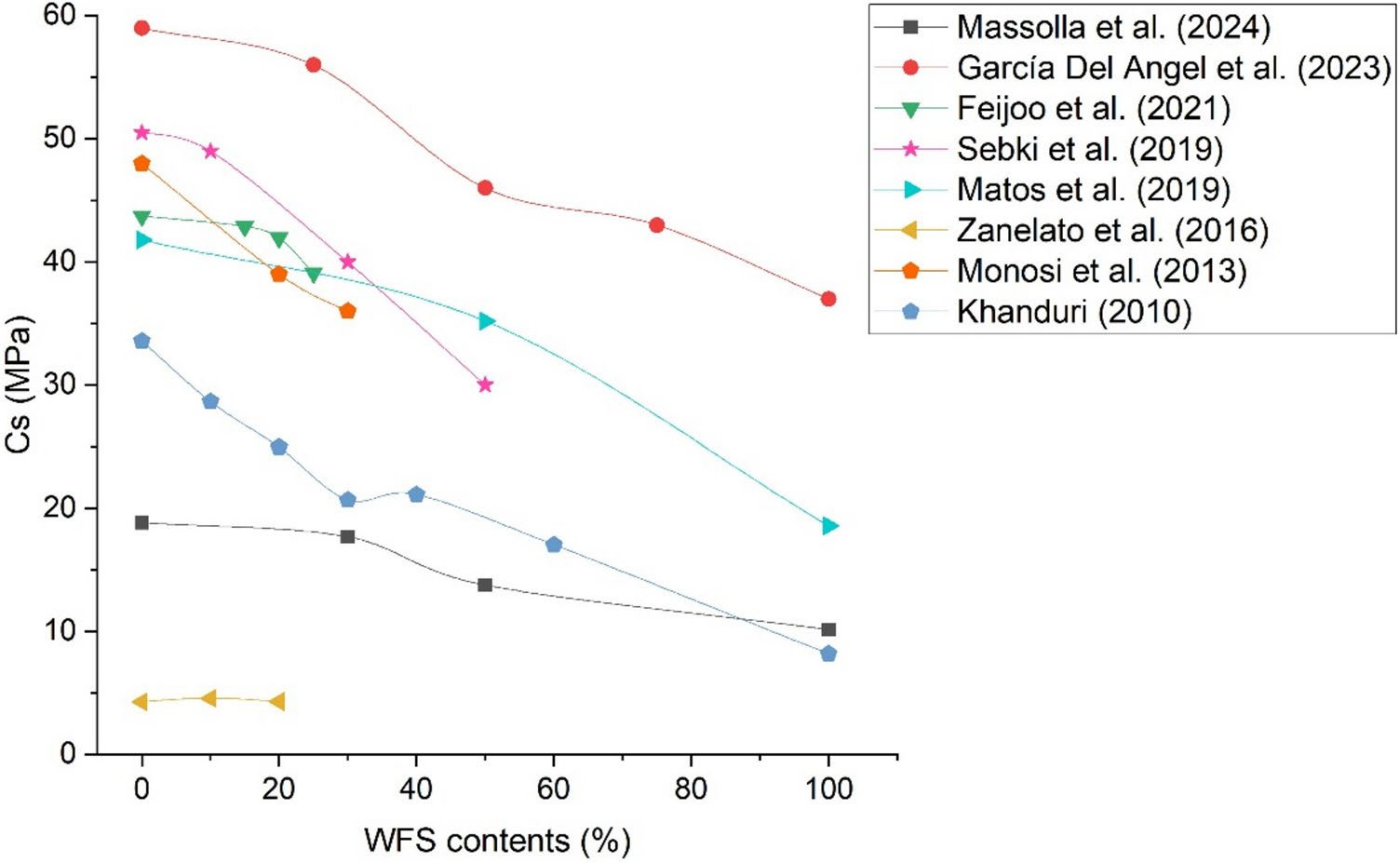
Behavior of cement mortars after 28 days of curing at different WFS levels: (a) compressive strength (CS); (b) flexural strength (FS)

To facilitate the visualization and analysis of the compressive strength (CS) behavior presented in Table 3, the graph shown in Fig. 6 was developed, displaying CS values as a function of the waste foundry sand (WFS) replacement levels reported by different authors. To ensure a more consistent comparison among the studies, only investigations employing green waste foundry sand and cement as the primary binder were considered. It is important to note that the study by Thamaraiselvi et al. (2020) was excluded from this comparative analysis because Ground Granulated Blast Furnace Slag (GGBFS) and Silica Fume were incorporated into the mixtures. The presence of these supplementary cementitious materials can significantly influence the mechanical performance of mortars, making it difficult to attribute the observed effects solely to the incorporation of WFS and, therefore, hindering a fair comparison among the selected studies.

In addition to the selection of studies with similar binder compositions, another factor that significantly influences the analysis of mortar performance is the application of WFS pretreatment methods, such as washing or calcination. Previous studies have reported that these treatments can increase the specific surface area of the waste and reduce its porosity, leading to improvements in mechanical properties and enabling the use of higher natural sand replacement levels (Matos et al. 2019; Sabour et al. 2022). However, information regarding the sustainability of these treatment processes remains limited, as their environmental and energy-related impacts have not been adequately assessed. In this context, further studies employing tools such as Life Cycle Assessment (LCA) and circular economy indicators are needed to quantify the benefits and impacts associated with these pretreatment techniques (Paul et al. 2022). Therefore, to ensure a more consistent comparison among the results reported in the literature, the analyses presented in the following sections consider only studies that used WFS without any pretreatment. 

Similarly, the type of foundry sand employed may also affect the mechanical behavior of mortars and, consequently, the comparability of the reported results. Therefore, only studies involving green foundry sand were considered in the subsequent analyses. Since the objective of this review is not to compare the mechanical behavior of mortars produced with different types of foundry sand, studies using chemically bonded foundry sands were excluded. This decision was made because the characteristics of chemically bonded sands differ substantially from those of green foundry sands, potentially affecting the mechanical performance of mortars and introducing an additional source of variability into the analysis.

According to Table 3 and the Fig. 6, the mixtures used mostly present high cement consumption, resulting in compressive strength values above what is recommended for the usual mortars (laying and coating). Furthermore, it is evident that, even among studies using the same type of WFS (green foundry sand), cement type, and evaluating specimens at the same curing age (28 days), significant variations in compressive strength are observed at identical WFS replacement levels. This finding highlights the influence of additional factors, such as the characteristics of the WFS, mixture proportions, water-to-cement ratio, specimen geometry and dimensions, as well as differences in testing procedures, all of which can affect the mechanical performance of the mortars.

### Fresh state of cement mortars produced with WFS

One of the most important aspects of conventional mortars (laying and coating) is workability, which must also be maintained in mortars with waste incorporation. This property is linked to the water/binder ratio, the texture of the fine aggregate, and the water holding capacity of the mixture. In this context, García Del Angel et al. (2023) report that as the WFS content in the mortars increases, the workability decreases, which leads to the addition of water in the mixture and, thus, an increase in the water/binder ratio and a reduction in mechanical properties (Monosi et al. 2013; Feijoo et al. 2021; Paul et al. 2022). This behavior can be justified by the physical characteristics of WFS (uniform grain size curve and lower FM), in addition to the presence of bentonite and coal (greater surface area, porosity, and water absorption) (Monosi et al. 2013; Navarro-Blasco et al. 2013; Paul et al. 2022).

The fineness modulus (FM) directly affects mortar workability, with lower FM values corresponding to higher specific surface areas and, consequently, greater water demand. Higher water demand generally requires an increase in the w/c ratio, which may lead to increased porosity and lower compressive strength in the hardened state (Monosi et al. 2013; García Del Angel et al. 2023). Moreover, according Carasek (2017), very fine particles impair adhesion with the cement paste, reducing the internal cohesion of the mortar.

García Del Angel et al. (2023) conducted a study to investigate the influence of the water/cement ratio on the mechanical properties of mortars, as well as the effect of WFS on workability. The authors reported that the incorporation of WFS may lead to two opposite effects: on the one hand, the particle geometry can improve mixture workability; on the other hand, the higher water absorption of WFS requires an increased water content to maintain adequate workability.

The reduction of the water/binder ratio (≤ 0.5) can contribute to improving the performance of mortars with WFS (Monosi et al. 2013; Vázquez-Rodriguez et al. 2018; García Del Angel et al. 2023). However, this reduction may negatively affect fresh-state performance, particularly during application (coating or masonry laying), often requiring the use of mineral additions or additives (water incorporator and/or water retained).

In this context, several studies (Navarro-Blasco et al. 2013; Feijoo et al. 2021) suggest replacement levels between 10 and 30% as a way to balance mechanical performance and fresh-state properties in WFS mortars. To preserve workability, different approaches have been reported in the literature: Monosi et al. (2013) incorporated a superplasticizer, while Sabour et al. (2022) improved workability by washing the WFS before mixing, thereby removing finer particles that increase water demand. Other authors (Zanelato et al. 2016; Sebki et al. 2019; García Del Angel et al. 2023; Massolla et al. 2024), adjusted the water-to-cement ratio according to the WFS replacement level. This approach compensates for the increased water demand associated with the substitution of natural sand, ensuring adequate workability across different mortar mixtures while maintaining consistent fresh-state behavior.

### Influence of specimen size and geometry on mechanical properties

Specimen size and geometry can influence the mechanical behavior of cementitious materials. In this context, some authors evaluate these parameters in cementitious materials (Fládr and Bílý 2018; Rawat et al. 2024). Rawat et al. (2024) reported that compressive strength generally decreases with increasing specimen size, which was attributed to the smaller fracture process zone in small specimens and their greater energy absorption capacity. For conventional concrete, cube specimens tend to exhibit higher compressive strengths due to the restraint of lateral expansion by the loading plates. In contrast, slender specimens are additionally affected by the length effect, resulting in a greater reduction in strength. However, this phenomenon was found to be less pronounced in high-strength engineered cementitious composite (HSECC) owing to its stronger interfacial transition zone and more homogeneous microstructure, resulting from the incorporation of very fine aggregates (< 300 μm).

In this context, given the fine particle size of WFS, which commonly contains particles smaller than 300 μm, it is possible that the mortars produced by some authors developed a denser, more homogeneous matrix, similar to that observed in HSECC. As a result, these mortars may have been less influenced by specimen size and geometry effects than is typically observed in conventional concrete.

### Analysis of compressive strength reductions as a function of WFS replacement level

**Fig. 7:**
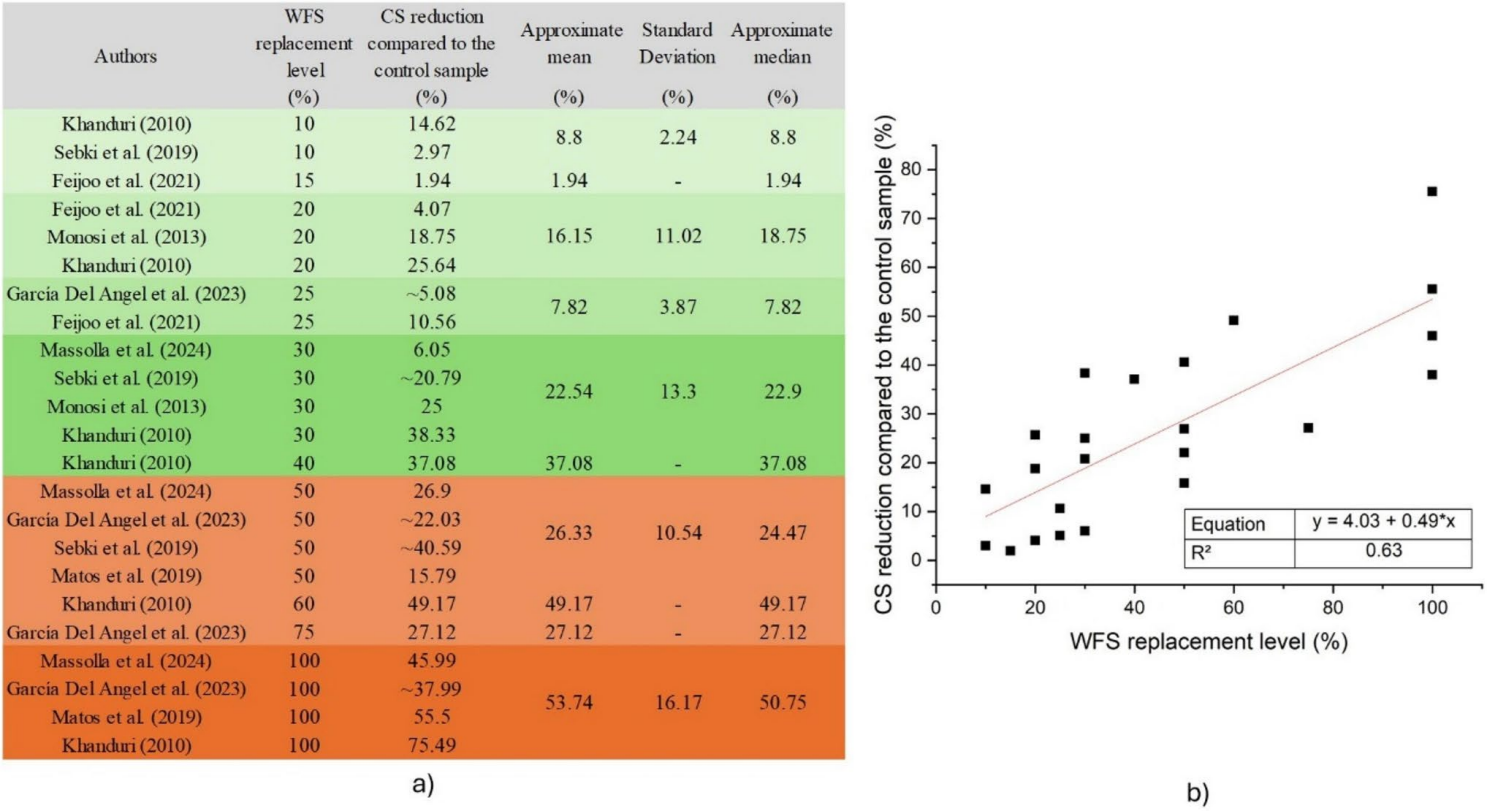
Effect of WFS replacement level on the compressive strength of mortars after 28 days of curing: a compressive strength reductions reported in the literature and b linear regression model relating WFS replacement level to compressive strength reduction based on the data presented in Figure 7a

Based on the results presented in Fig. 6, a summary table was developed (Fig. 7a), compiling the percentage reductions in compressive strength reported for each WFS replacement level relative to the corresponding control mixtures. Subsequently, the values presented in this table were used to generate a correlation plot (Fig. 7b), allowing for a more detailed assessment of the relationship between WFS content and compressive strength performance across the selected studies. It should be noted that the results reported by Zanelato et al. (2016) were not included in the summary table or in the correlation analysis because the incorporation of WFS produced only negligible changes in the mechanical performance of the mortars. As shown in Fig. 6, the mixtures exhibited only minor variations in compressive strength over the investigated replacement levels. In fact, a slight increase in compressive strength was observed at a 10% WFS replacement level compared to the reference mixture. This behavior may be attributed to the filler effect provided by the finer WFS particles, which tend to fill the voids within the cementitious matrix, reducing porosity and resulting in a denser and more compact microstructure (García Del Angel et al. 2023).

As shown in Fig. 7a, considerable variations in compressive strength reductions were observed among studies reporting the same WFS replacement level. Among the replacement levels evaluated, 10% and 25% exhibited the lowest standard deviations. These variations are discussed in the following sections, with the analysis organized according to the different WFS replacement levels.

### Comparison between authors employing 10 and 15% replacement levels

Some authors (Khanduri 2010; Sebki et al. 2019; Feijoo et al. 2021) analyzed low levels of WFS in mortars (between 10 and 15% of WFS), showing the compressive strength behavior.

Therefore, it can be observed that Sebki et al. (2019) and Khanduri (2010) reported markedly different reductions in mechanical properties despite adopting the same WFS replacement level of 10%. Khanduri reported lower compressive strength values and a greater reduction relative to the control mixture than Sebki et al. (2019). This difference may be primarily attributed to the higher cement content used by Sebki et al. (2019), who adopted a cement-to-sand ratio of 1:2, whereas Khanduri (2010) employed a ratio of 1:3. The higher cement content likely promoted the formation of a greater amount of hydration products, resulting in higher compressive strength values. Furthermore, for the same replacement level, the mixture of Sebki et al. (2019) contained a larger proportion of cement relative to foundry sand than the mixture used by Khanduri (2010), which may have mitigated the adverse effects of WFS incorporation and contributed to the smaller reduction in mechanical performance.

In addition, the specimen dimensions may also have contributed to the lower compressive strength values reported by Khanduri (2010). While both studies employed cubic specimens, Khanduri (2010) used larger cubes than Sebki et al. (2019). As previously discussed, an increase in specimen size generally leads to lower measured compressive strength values due to the size effect and differences in the fracture process zone. Therefore, the larger specimen dimensions adopted by Khanduri (2010) may have further contributed to the lower compressive strength values observed in comparison with those reported by Sebki et al. (2019).

Differences in the fineness modulus (FM) of the WFS may also have influenced the results. Sebki et al. (2019) and Khanduri (2010) reported FM values of 2.47 and 1.91, respectively. As discussed in the fresh-state analysis, finer WFS particles tend to increase water demand, which may partially explain the lower compressive strength observed by Khanduri (2010). Although slight differences in specimen dimensions were observed, both studies adopted cubic specimens. Therefore, it is believed that cement content, FM, and w/c ratio were the main factors responsible for the differences in the percentage reductions relative to the control mixtures. Furthermore, Khanduri et al. cured the specimens at a higher temperature than that reported in most of the other studies (27 °C), with considerable temperature variation. This factor may have influenced the greater reductions in compressive strength observed with increasing WFS content, since higher curing temperatures can promote shrinkage and microcracking, thereby affecting the mechanical performance of the mortars.

A comparison between the studies conducted by Sebki et al. (2019) and Feijoo et al. (2021) shows that both authors used similar WFS replacement levels (10% and 15%), resulting in small and comparable reductions in mechanical properties (below 3%). However, Feijoo et al. (2021) reported a lower reduction relative to the reference mixture, despite using a leaner mix proportion (1:2.75), a 5% higher WFS content, and a sand with a lower FM. This result suggests that the 15% WFS incorporation may have provided a more favorable particle packing condition, thereby minimizing the loss of mechanical performance.

### Comparison between authors employing 20 and 25% replacement levels

The difference in cement content between the studies may also explain the differences in both the compressive strength values and the reductions observed at a 20% WFS replacement level. Despite using the same replacement percentage, Khanduri (2010) reported a compressive strength reduction of 25.64%, whereas Feijoo et al. (2021) and Monosi et al. (2013) observed reductions of only 4.07% and 18.75%, respectively. The water-to-cement ratio may also have contributed to the contrasting reductions in compressive strength observed between Khanduri (2010) and Feijoo et al. (2021). However, this parameter could not be reliably compared because the study of Khanduri (2010) presents inconsistent information regarding the water-to-cement ratio. Specifically, a value of 0.54 is reported in the text, whereas the mixture proportions provided in the corresponding table result in a calculated water-to-cement ratio of approximately 0.46. This discrepancy makes it difficult to determine the actual ratio adopted and, therefore, to evaluate its effect on the reported mechanical performance.

Between Monosi et al. (2013) and Feijoo et al. (2021), it is believed that one of the reasons for the variations in mechanical behavior may be the difference in cement content, even though both studies adopted the same water-to-cement ratio and still exhibited notable differences in compressive strength results. In addition, workability could not be directly compared, since Feijoo et al. (2021) did not report flow table values. It is also suggested that specimen geometry and size were major factors influencing the differences observed among Monosi et al. (2013), Feijoo et al. (2021), and Khanduri (2010). While Feijoo et al. (2021) and Khanduri (2010) used cubic specimens, Monosi et al. (2013) employed prismatic specimens, which may have significantly influenced the measured mechanical performance.

In this context, among these three studies, Feijoo et al. (2021) reported the highest compressive strength values and the smallest reductions associated with WFS incorporation. This behavior may be partially attributed to the smaller cubic specimens adopted by these authors. As previously discussed, cubic specimens generally tend to yield higher compressive strength values than prismatic specimens due to confinement effects generated by the loading plates. Furthermore, smaller specimens often exhibit higher measured compressive strengths than larger ones as a result of the size effect, which is associated with a smaller fracture process zone and a greater capacity for energy absorption prior to failure. Consequently, the specimen geometry and dimensions adopted by Feijoo et al. (2021) may have contributed to the higher compressive strength values and lower strength reductions observed in comparison with Monosi et al. (2013) and Khanduri (2010).

Regarding specimen geometry, the compressive strength of the prismatic mortar reported by García Del Angel et al. (2023) (~ 56 MPa) was higher than that reported by Feijoo et al. (2021) (39.11 MPa), despite both studies evaluating a similar WFS replacement level (25%). This behavior contrasts with the trend discussed in the previous paragraph, in which prismatic specimens generally exhibited lower compressive strength values than cubic specimens, as reported for cement-based composites in the literature (Fládr and Bílý 2018; Rawat et al. 2024). Therefore, specimen geometry alone is insufficient to account for the differences observed between the studies.

Other parameters likely exerted a greater influence on the mechanical performance, including mixture proportioning, workability, and fineness modulus (FM). Although both studies adopted similar water-to-cement ratios, García Del Angel et al. (2023) omitted information regarding the mortar mix proportions and the FM of the WFS, whereas Feijoo et al. (2021) lacked data on flow table results. Consequently, the influence of these parameters cannot be directly assessed or compared between the two studies.

Regarding the study conducted by Feijoo et al. (2021), it is important to highlight that the curing method may have been a significant factor influencing the observed results. Among the studies analyzed, these authors were the only ones to cure the specimens by immersion in lime-saturated water. This condition may have enhanced the self-healing process of the mortars, since the precipitation of calcium hydroxide within the cracks contributes to their filling and reduces permeability. As a result, the mortars evaluated by Feijoo exhibited the smallest reductions in compressive strength at all replacement levels investigated.

### Comparison between authors employing 30 and 40% replacement levels

As the WFS replacement level increased to 30% and 40%, the studies presented in Fig. 7a that evaluated these replacement levels reported more pronounced reductions in compressive strength relative to the reference mixture, ranging approximately from 20 to 38%. The main exception was Massolla et al. (2024), who observed a reduction of only 6% at a 30% WFS replacement level.

The comparison between the results reported by Massolla et al. (2024) and those of the other studies should be approached with caution, as these authors employed a mix proportion with lower cement content (1:6). Consequently, for the same replacement rate, the amount of WFS relative to the cement content was likely higher than in the other studies, potentially improving particle packing. This behavior suggests that a 30% WFS replacement level may be close to the optimum level for mortars with lower cement consumption.

Furthermore, although Massolla et al. (2024) reported the lowest absolute compressive strength values among the studies analyzed, the reductions associated with WFS incorporation remained limited, reaching only 6% at a 30% replacement level, even with a relatively high water-to-cement ratio. This finding suggests that the magnitude of strength loss associated with WFS incorporation is not always directly related to the absolute strength of the reference matrix, indicating that factors other than the water-to-cement ratio may also exert a significant influence on this behavior.

This hypothesis is further supported by the findings of García Del Angel et al. (2023) and Feijoo et al. (2021), who despite employing mixtures with higher cement contents, also reported strength reductions below 11% at a similar replacement level (25%), indicating that higher WFS replacement levels do not necessarily result in significant losses in compressive strength.

Among the remaining studies, Sebki et al. (2019) and Khanduri (2010) exhibited, at a 30% WFS replacement level, a trend similar to that previously observed at 10% replacement, as the differences between their reported strength reductions remained relatively consistent with those observed at lower replacement levels. In contrast, Monosi et al. (2013) and Khanduri (2010), who had exhibited similar reductions in compressive strength at a 20% WFS replacement level, showed differences exceeding 10% when the WFS replacement level was increased to 30%.

The divergence observed between the results of Monosi et al. (2013) and Khanduri (2010) at a 30% WFS replacement level suggests that the factors previously discussed, including cement content, WFS FM, and specimen geometry, exerted an even greater influence on the mechanical performance of the mortars as the WFS replacement level increased. Furthermore, the lower water-to-cement ratio adopted by Sebki et al. (2019) (0.351), compared with that used by Khanduri (2010), may have contributed to the development of a denser matrix at this replacement level. This same factor, combined with the higher cement content and the use of cubic specimens, may also explain the smaller strength reductions observed by Sebki et al. (2019) relative to Monosi et al. (2013).

Regarding the study conducted by Khanduri (2010), it is important to highlight that the authors adopted water immersion curing, a procedure also used by Sebki et al. (2019), Monosi et al. (2013), and Feijoo et al. (2021). Considering that water immersion promotes the continuous hydration of cement and may contribute to the preservation of mechanical properties, compressive strength reductions similar to those reported in these studies would be expected. However, Khanduri (2010) reported the highest compressive strength losses at all replacement levels investigated. This finding suggests that other factors had a more significant influence on the mechanical behavior of the mortars, particularly the curing temperature conditions. The study was conducted at 27 °C, a temperature higher than that adopted by the other authors, and also presented a temperature variation of ± 20 °C, which may have affected the hydration process and the development of the mortar microstructure.

Regarding relative humidity, this parameter could not be considered in the comparative analysis, since not all studies reported the humidity conditions during curing. Consequently, the lack of consistent data prevented a reliable assessment of the influence of relative humidity on the differences observed in the mechanical performance of the mortars.

### Comparison between authors employing 50 to 100% replacement levels

For Sebki et al. (2019), increasing the WFS replacement level to 50% resulted in compressive strength reductions that were nearly twice those observed at a 30% replacement level, highlighting a more pronounced influence of waste foundry sand on the mechanical performance of mortars at high replacement levels. This behavior may be associated with the lower workability of the mixtures (180 mm) compared with the other studies that evaluated similar replacement levels, for which flow values ranged from approximately 200 to 262 mm, as reported by Massolla et al. (2024) and Matos et al. (2019)

Both Sebki et al. (2019) and Khanduri (2010) maintained a constant water-to-cement ratio, which led to a progressive reduction in workability as the WFS replacement level increased. This reduction in consistency may have contributed to the more pronounced strength losses observed at higher replacement contents. It is also noteworthy that these authors, despite using mixtures with relatively higher cement contents, reported some of the largest decreases in compressive strength. Specifically, these results correspond to mixtures incorporating 50% WFS in the case of Sebki et al. (2019) and 60% in the case of Khanduri (2010)

In contrast, Matos et al. (2019) reported a smaller reduction in compressive strength at the 50% replacement level than Sebki et al. (2019). This difference may be partly related to the use of cylindrical specimens, which can result in higher compressive strength values. Moreover, Matos et al. (2019) used a WFS with an FM of 1.0. Similarly, Massolla et al. (2024) also reported a smaller reduction in compressive strength than Sebki et al. (2019) at the same replacement level, employing a WFS with an FM of 1.2. These results indicate that lower FM values may contribute to improved compressive strength performance in mortars containing high levels of WFS. In this context, the findings of Matos et al. (2019) and Massolla et al. (2024) suggest that, at high replacement levels, the use of WFS with a lower FM may be beneficial, provided that adequate workability is maintained. This behavior may be associated with improved particle packing, thereby mitigating some of the negative effects resulting from the increased WFS content.

A similar trend can be observed for mortars produced with 100% WFS. Massolla et al. (2024) and Matos et al. (2019), who used materials with lower FM values, reported less pronounced reductions in compressive strength. Notably, García Del Angel et al. (2023) reported the smallest strength reduction at a 100% WFS replacement level and also observed relatively low reductions at 75% and 100% replacement, suggesting favorable mechanical performance even at high WFS contents. Nevertheless, the absence of information regarding both the WFS FM and the mortar mix proportions prevents a direct comparison with the other studies.

Based on the individual compressive strength reduction values reported by each author, relative to the corresponding control mortar and at different WFS replacement levels (Fig. 7a), a linear correlation was established to evaluate the relationship between WFS replacement level and compressive strength reduction. The resulting correlation (Fig. 7b) indicates that, in general, the studies reported in the literature tend to show progressive reductions in compressive strength as the WFS replacement level increases. For replacement levels of 10%, 20%, 30%, 50%, and 100%, the median compressive strength reductions were 8.8%, 18.75%, 22.9%, 24.47%, and 50.75%, respectively. These results demonstrate a general trend of increasing strength loss with increasing WFS content.

However, the coefficient of determination (*R*^2^) of 0.63 indicates considerable variability among the different studies. In other words, although a general trend of decreasing compressive strength with increasing WFS replacement can be observed, the magnitude of this reduction varies substantially across authors. This variability may be attributed to the factors discussed previously, including differences in material characteristics, mix proportions, specimen geometry, and curing procedures. Therefore, these findings reinforce that, due to the heterogeneity and limitations of the available literature, defining an optimum WFS replacement level remains a challenge.

### Analysis of flexural strength reductions as a function of WFS replacement level

**Fig. 8:**
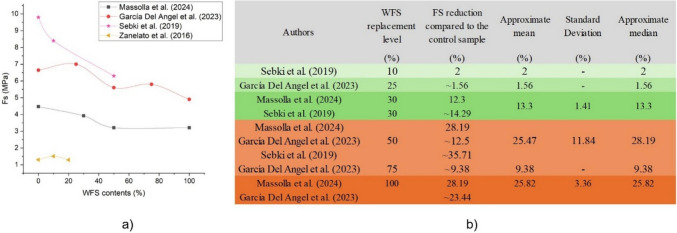
Influence of WFS content on the flexural strength (FS) of mortars after 28 days of curing: a nominal FS values and b percentage reduction in FS relative to the reference mortar

Flexural strength is an equally important property for mortars. For this reason, the same methodological approach adopted for the compressive strength table was also applied to flexural strength, selecting studies that used green sand and reported results at 28 days of curing. Thus, Fig. 8 presents the FS values and an analysis of the reduction in FS of mortars containing WFS in relation to the reference mortars.

When evaluating Fig. 8, it is observed that flexural strength (FS) is less investigated in the literature compared to compressive strength (CS). Furthermore, it can be seen that strength reductions show wide variation among authors and across different WFS content levels. Nevertheless, similar behaviors can be identified between CS and FS, particularly the trend of strength reduction with increasing WFS content. Additionally, mortars tend to exhibit smaller reductions in FS relative to the reference mixture at low replacement levels; however, this difference decreases as the WFS content increases, with a more pronounced loss of mechanical performance at higher substitution levels.

Despite the similarities observed, based on analyses of CS and FS reported by different authors who evaluated both properties, it was possible to verify that FS is, in most cases, less affected by the incorporation of WFS than CS. This is evidenced by the fact that, in general, the percentage reductions in FS of mortars containing WFS, relative to the reference mixture, are lower than those observed for CS (Sebki et al. 2019; García Del Angel et al. 2023; Massolla et al. 2024).

This behavior becomes even more evident in the results reported by García Del Angel et al. (2023) where, at a 75% replacement level, a reduction of approximately 27.12% was observed in CS, while FS showed a reduction of only 9.38%. On the other hand, Massolla et al. (2024) at replacement levels of 30% and 50%, they reported more pronounced reductions in FS than in CS. This discrepancy may be associated with several factors, such as the WFS batch, cement type, mineral additions, use of admixtures, material proportioning, and the experimental methodology adopted (specimen type, curing time, and curing conditions).

These differing behaviors indicate variability in flexural strength results in the literature, highlighting that this property still requires further investigation. In particular, there is a lack of studies providing direct and systematic comparisons between CS and FS in mortars containing WFS, which limits a more consistent understanding of their mechanical behavior.

It is noteworthy that a representative linear correlation could not be established, as the coefficient of determination was only *R*^2^ = 0.32. This low value reflects the high variability of the available data, which may be attributed to the limited number of studies investigating this property. Moreover, substantial differences in the reported reduction percentages were observed even among studies evaluating the same WFS replacement levels, hindering the identification of a consistent relationship between the variables.

### Other relevant properties of mortars

Although compressive strength (CS) and flexural strength (FS) are the mechanical properties most frequently reported in the literature, other properties are equally important for assessing mortar performance. Among these, adhesion strength deserves particular attention; however, it has been investigated by only a few authors (Massolla et al. 2024). Thus, when analyzing this property of mortars with WFS in the contents of 0% (reference), 30%, 50%, and 100%, they obtained results of 1.67 MPa, 1.42 MPa, 1.11 MPa, and 0.82 MPa, respectively. This indicates that the addition of WFS caused reductions between 15 and 50% compared to the reference trace (0% WFS). The authors attributed these reductions, relative to the control mortar, to the lower rate of cement hydration reactions in the presence of WFS. This led to a lower proportion of hydrated particles and, consequently, impaired the formation of adhesive bonds between the mortar and the substrate. Another justification for adhesion strength reduction is related to the WFS grains, which, due to their sub-angular to rounded shape, result from the wear of the casting process (FIRST 2004), have a low roughness index, and do not contribute positively to adhesion strength.

The static (E) or dynamic (Ed) modulus of elasticity is also an important mechanical property of coating mortars. This parameter is very important, since it indicates the higher or lower rigidity of mortars, especially for coating ones. According to NBR 13281-1 (ABNT 2023), coating mortars should not have Ed values above 14 GPa, as the greater rigidity hinders their functionality. However, there are few studies on this property in mortars with WFS (Paul et al. 2022; Massolla et al. 2024) determined the Ed of mortars with 30%, 50%, and 100% WFS, and the results were 22, 20, and 17 GPa, respectively. Thus, compared to the mortar without WFS (24.59 GPa), Ed presents reductions of 9%, 16%, and 30% for the WFS contents of 30%, 50%, and 100%, respectively. Paul et al. (2022) analyzed the E of mortars with 30% and 100% WFS content, where the values were 33.9 and 18.2, with reductions of 7% and 50%, respectively. The results did not indicate significant reductions up to the 30% WFS content, indicating that the content is better. It is noteworthy that these studies were developed with mortars in unusual proportions of their components, which makes it difficult to analyze such results for practical applications.

## General discussion

From the analysis of the results available in the literature, it is observed that mortars with low levels of natural sand replacement by WFS tend to exhibit smaller reductions in compressive strength when produced with WFS of higher fineness modulus (FM), higher cement content, and cured by immersion in lime-saturated water. In addition, the studies reporting the lowest strength losses used cubic specimens, suggesting that specimen geometry may also have influenced the obtained results (Rawat et al. 2024).

In general, the results indicate that, for replacement levels below 50% WFS, the mortars may have benefited from the higher FM of the material, resulting in lower losses of workability when compared to mortars produced with finer WFS. Furthermore, within this replacement range, mixtures with higher cement contents tend to exhibit higher compressive strength (CS) values and smaller reductions in this property relative to the reference mortar.

On the other hand, for replacement levels above 50%, a different behavior is observed. Unlike what is seen at lower replacement levels, mortars with lower cement content exhibited the smallest reductions in compressive strength. This result suggests that, at high substitution levels, cement content is no longer the dominant factor in mechanical performance, while the FM of the WFS becomes more influential. This is because the mortars showing the lowest strength losses used WFS with a lower FM.

Thus, for high replacement levels (> 50%), it is believed that the beneficial effect of WFS on particle packing played a more relevant role than the increase in cement content. It should be noted that the studies reporting the lowest reductions in compressive strength maintained flow table values between 200 and 260 mm, indicating that maintaining adequate workability levels may also have contributed to the improved mechanical performance observed; for this purpose, some authors adjusted the water-to-cement ratio (Massolla et al. 2024).

Still regarding high replacement levels, it is also suggested that the cylindrical geometry of the specimens may have contributed to the lower reductions observed in compressive strength (CS), since the smallest strength losses were reported for mortars molded in this format. This result is consistent with the well-established trend in the literature that cylindrical specimens tend to exhibit higher compressive strength values compared to prismatic or cubic specimens (Rawat et al. 2024).

Given the current state of knowledge discussed in this section, the variability of results concerning the mechanical properties of mortars can also be justified by both properties of the fresh state and the physicochemical characteristics of WFS, as well as the variability of the FM that each batch of WFS can present, along with the type and consumption of cement used, mineral additions, curing time, curing method, water/cement ratio, and pretreatment performed in WFS (Andrade et al. 2018). Therefore, as each study uses WFS mixtures with different characteristics and variable methodologies, the results are usually not applicable and lack important statistical analyses to determine the ideal content. In addition, the few existing studies used mortars with unusual ratios between the materials, which prevents the interpretation of the data for practical applications (laying and coating). It is worth noting that this type of application can increase demand for WFS, given that mortar consumption is considerable and that it mostly does not serve a structural function. In this sense, there is a need for studies with the usual mixture of mortars, in which the ratio of the materials is compatible with their functionality, thus allowing the investigation of other important properties, including the modulus of elasticity, bond strength, and durability performance.

As discussed throughout this article, several parameters must be considered to enable a proper comparison of the mechanical properties of mortars containing WFS. However, this review identified important gaps in the literature that hinder more accurate comparisons among studies employing the same replacement levels.

Among the main limitations identified is the lack of information related to the fresh-state properties of mortars, particularly the water-to-cement (w/c) ratio and flow table test results. These parameters are essential, as they directly influence the microstructure and, consequently, the mechanical performance of mortars in the hardened state.

Furthermore, detailed information regarding the characteristics of the WFS used is often lacking in the literature. Important parameters such as the type of foundry sand, the cast metal or alloy associated with the manufacturing process, and the application of pretreatment procedures are not consistently reported. Likewise, key experimental variables, including specimen geometry and dimensions, curing method, curing temperature, and relative humidity, are frequently omitted or insufficiently described, limiting the reliability of comparisons among studies.

Another highly relevant parameter is the FM of the WFS, as this property significantly affects both the workability and mechanical performance of mortars. However, some authors report only the particle size distribution curves without directly providing the FM, making it difficult to establish consistent comparisons among different studies.

The lack of consistent reporting of curing conditions, including temperature and relative humidity, hindered meaningful comparisons among studies.

Therefore, future studies should seek to provide more comprehensive and standardized reporting of experimental parameters, thereby facilitating more reliable comparisons and improving the understanding of the influence of WFS on mortar performance.

## Conclusions

This article was aimed at conducting a systematic review on the mechanical behavior of mortars with WFS, identifying the factors that influence the mechanical strength of these mortars and the gaps in the literature that hinder consensus on the appropriate replacement content for natural sand. By the literature search, the following conclusions were obtained:

According to the results obtained in the analyzed studies, most of the authors consulted reported reductions in the mechanical strengths of mortars with the addition of WFS, which worsen when the content increases; these reductions occur because of the inorganic binders present in WFS samples, which have a lower fineness modulus and more uniform grain size compared to common sand. This may require a higher water/binder ratio, leading to a loss of mechanical performance. However, in some cases, incorporating a low percentage of WFS to replace CS in mortars offers benefits, such as improving their durability by filling capillary pores.

Some authors perform pretreatment in WFS, such as calcination and washing; these processes remove the inorganic elements. However, it should be noted that these types of treatments may be unfeasible in the environmental and economic scenario.

Although several factors influence the mechanical properties of mortars containing WFS, making it difficult to define an optimal replacement level, the detailed analysis conducted in this study allowed the identification of general trends. Despite the variations in compressive strength (CS) and reduction levels observed in mortars containing WFS, the results indicate consistent patterns as a function of the replacement level. For substitution levels between 10 and 25%, reductions relative to the reference mortars ranged approximately from 2 to 26%. At around 30% replacement, most of the analyzed studies reported reductions between 20% and 38.33%, although one study reported a much lower reduction of only 6%, highlighting the possibility of limited strength loss even at relatively high replacement levels.

At 50% replacement, reductions ranged approximately from 15 to 40%, while for full replacement (100%) more pronounced losses were observed, ranging from 38 to 75%. Overall, it can be concluded that increasing WFS content generally leads to higher reductions in compressive strength; however, the magnitude of this effect strongly depends on the material characteristics, mix design, and testing conditions adopted in each study.

To maintain the mechanical performance, many authors increase the consumption of the binder; they did not study the mixture for application purposes, using a dosage of 1:3 (cement/sand), resulting in high mechanical strength values (FS and CS) even with the respective reductions caused by the presence of WFS. Thus, if further studies focus on studying mechanical behavior considering the applicability of the material, they may enable higher levels of WFS mortars, because the function of coating and laying mortars does not always require high values of mechanical strength.

Although WFS causes a loss of strength in mortars, large volumes of this residue can be used in this material, which is in high demand in the construction industry, mainly for nonstructural applications.

In this sense, the first stage of a study on the use of WFS as a fine aggregate is to define the optimal replacement content depending on the desired properties and characteristics of the batch of waste. We also highlight the importance of defining this ratio for cement materials with common aggregate and thus obtain a better packing of the grains and filling of the capillary pores, favoring mechanical strength and durability. This will contribute to knowledge about the properties and performance of laying and coating mortars.

Nevertheless, there is still a gap in the literature of WFS mortars with the use of the usual mixture for application purposes. Finally, it would be interesting to establish similar conditions for mortars in the next studies, to reduce the variables that influence the mechanical behavior of mortars with WFS, which generate data variability and controversial conclusions about this material, in addition to analyzing their performance and durability.

## Future research suggestions

**Fig. 9:**
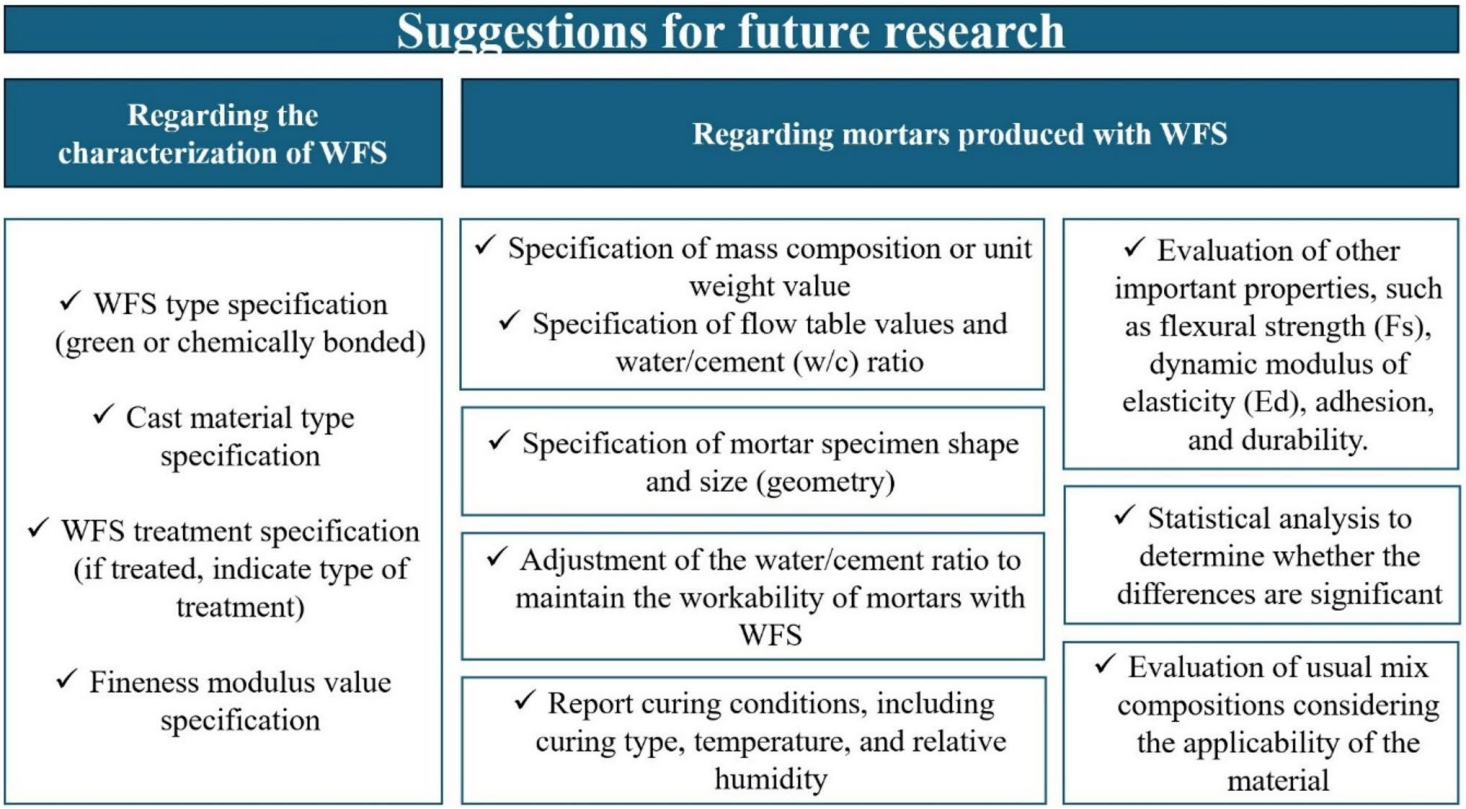
Compilation of key recommendations for future research

The lack of some essential information in the literature makes it difficult to compare the mechanical behavior of mortars produced by different authors. In addition, research gaps were identified that limit the advancement of the applicability of mortars containing WFS. In this context, a compilation of parameters that should be considered in future studies was developed and is presented in Fig. 9, which may serve as a checklist for subsequent investigations.

The application of the guidelines presented in Fig. 9 has the potential to enhance the development and applicability of mortars containing WFS. In addition, it may contribute to reducing the variability of mechanical properties for the same substitution level, making comparisons between studies more consistent, reproducible, and reliable.

## Data Availability

The authors confirm that all data generated or analyzed during this study are included in this published article.
